# Maternal consumption of urbanized diet compromises early-life health in association with gut microbiota

**DOI:** 10.1080/19490976.2025.2483783

**Published:** 2025-04-02

**Authors:** Rong Huang, Guicheng Zhou, Jie Cai, Cha Cao, Zhenjun Zhu, Qingping Wu, Fen Zhang, Yu Ding

**Affiliations:** a Department of Food Science and Engineering, College of Life Science and Technology, Jinan University, Guangzhou, China; b Guangdong Provincial Key Laboratory of Microbial Safety and Health, State Key Laboratory of Applied Microbiology Southern China, Institute of Microbiology, Guangdong Academy of Sciences, Guangzhou, China

**Keywords:** Maternal urbanized diet, early-life health, gut microbiota, breast milk, metabolome, transmission

## Abstract

Urbanization has significantly transformed dietary habits worldwide, contributing to a globally increased burden of non-communicable diseases and altered gut microbiota landscape. However, it is often overlooked that the adverse effects of these dietary changes can be transmitted from the mother to offspring during early developmental stages, subsequently influencing the predisposition to various diseases later in life. This review aims to delineate the detrimental effects of maternal urban-lifestyle diet (urbanized diet) on early-life health and gut microbiota assembly, provide mechanistic insights on how urbanized diet mediates mother-to-offspring transfer of bioactive substances in both intrauterine and extrauterine and thus affects fetal and neonatal development. Moreover, we also further propose a framework for developing microbiome-targeted precision nutrition and diet strategies specifically for pregnant and lactating women. The establishment of such knowledge can help develop proactive preventive measures from the beginning of life, ultimately reducing the long-term risk of disease and improving public health outcomes.

## Introduction

Urban living offers pregnant women easy access to comprehensive medical advice, nutritional supplements, and professional dietary guidance. Nonetheless, urbanization also introduces dietary changes characterized by increased consumption of total energy, saturated fat, animal proteins, refined grains, added sugar, sodium and food additives, alongside inadequate intake of fermentable fibers and phytonutrients.^1^ These dietary shifts throughout pregnancy and lactation are linked to adverse health outcomes in their offspring, including low birth weight,^2^ intestinal inflammation,^3^ and neurodevelopmental disorders,^4^ although specific mechanisms by which these dietary components in urban diets impact offspring health remain unclear. Concurrently, recent findings increasingly highlight urbanized dietary patterns significantly alter gut microbiota, promoting the accumulation of detrimental metabolites such as trimethylamine (TMA)^5^ and the depletion of favorable metabolites like short-chain fatty acids (SCFAs) and microbial indole metabolites.^6^ These metabolic disturbances are linked to impaired metabolic or immune functions, thereby contributing to the increasing global prevalence of non-communicable diseases (NCDs).^7,8^ Notably, emerging evidence indicates that some of these metabolites can be vertically transmitted from the mother to offspring and play essential regulatory roles in early life development.^9^ This vertical transmission may represent a key mechanism through which maternal dietary patterns exert long-term effects on offspring health.

However, these scattered findings have not yet been integrated into a cohesive framework to explain how maternal urbanized diet influences offspring health. Therefore, this review aims to systematically consolidate existing evidence to highlight the adverse effects of urbanized diet and provide detailed mechanistic insights into how these diets alter maternal gut microbiota, thereby impeding the normal transmission of bioactive signals to offspring, affecting the health and developmental trajectories of offspring. On this basis, we propose future research directions and hypothetical frameworks to address existing knowledge gaps, thereby enhancing our understanding and response to health challenges that maternal urbanized diets pose to offspring. This knowledge will inform the development of microbiome-targeted precision nutrition strategies for pregnant and lactating women, to enhance maternal and offspring health, thereby advancing public health and well-being.

## Maternal consumption of urbanized diet affects offspring’s health outcomes and gut microbiome assembly

### The rising global diseases burden in early life under urbanization

**Figure 1.**
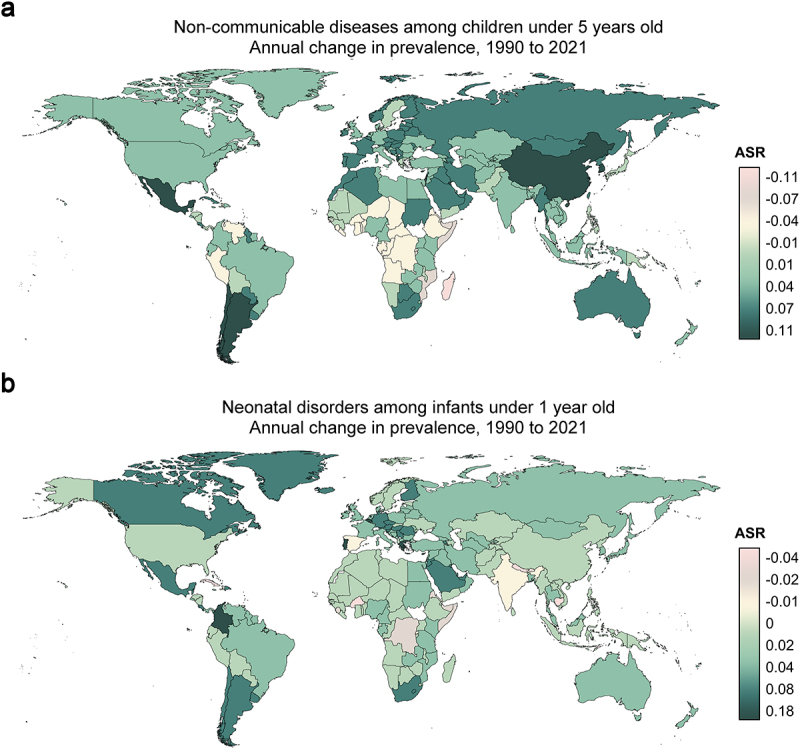
Global changes in disease burdens of infants and children with urbanization. (a) Changes in total prevalence (per 100,000) of 104 non-communicable diseases (NCDs) among children under 5 years across 203 countries between 1990 and 2021. (b) Changes in total prevalence (per 100,000) of neonatal disorders among infants under 1 year old across 203 countries between 1990 and 2021.

To elucidate the adverse effect of dietary transition during urbanization on the offspring’s health, we first evaluate the changes in the prevalence of disease in newborns and children (below 5 years old) from 1990 to 2021 (a period undergoing rapid urbanization) based on data from Global Burden of Disease Study (GBD) 2021 (https://vizhub.healthdata.org/gbd-compare/).^10^ Between 1990 and 2021, a global upward trend in neonatal disorders and non-communicable diseases (NCDs) among children under five years of age was observed across most nations (Figure 1(a,b)). Notably, rapidly urbanizing developing nations – particularly China, Mexico, Argentina, South Africa, and Colombia across four continents – showed marked increases in pediatric NCD prevalence (Figure 1a). Elevated neonatal morbidity rates were also observed in these regions, with Colombia exhibiting the most pronounced escalation (Figure 1b). Moreover, longitudinal epidemiological studies documenting substantial increases in inflammatory conditions such as asthma and inflammatory bowel disease among infants in the United States and other industrialized nations over recent decades, indicate potential urbanization-driven modifications to neonatal immune system development.^11–13^ Complementary analyses of urban-rural health disparities further reveal significant associations between urbanization and elevated risks of neonatal mortality^14^ and pediatric asthma,^15,16^ rhinitis,^17^ eczema,^18^ food allergy^19^ and obesity.^20^ The cumulative evidence underscores urbanization as an emerging environmental determinant of adverse offspring outcomes.

### Urban maternal nutrition consumption contributes to adverse outcomes in early life

Although factors influencing disease incidence are likely multifaceted, a growing body of evidence suggests that changes in dietary habits during urbanization may be a significant contributing factor. The process of urbanization has induced a gradual Westernization of dietary patterns, characterized by excessive intake of total calorie content and ultra-processed foods, which are enriched in saturated fats, animal proteins, and high levels of sugar and salt, coupled with a continual decline in dietary fiber consumption.^21^ Epidemiological studies have revealed that the elevated maternal consumption of saturated fats, added sugars, and sodium, which are common in the Western diet, is linked to an increased risk of gestational hypertension, obesity, preterm birth and low birth weight.^22,23^ On the contrary, the Mediterranean diet, a dietary pattern originated from agricultural societies and typified by higher consumption of vegetables, fruits, nuts, legumes, whole grains, fish, and olive oil, may lower the risk of maternal gestational hypertension, low birth weight and incidence of asthma in newborns.^24–27^ The evidence presented above illustrates that urbanization-induced alterations in maternal diet may engender detrimental impacts on both maternal and offspring health. In this context, extensive research is now dedicated to elucidating the mechanisms by which specific food ingredients within contemporary urban diets contribute to these adverse multigenerational effects. For example, maternal consumption of high-fat and low-fiber foods is associated with poor fetal growth, underdeveloped immune system during early life, and increased susceptibility to metabolic and neurological disorders in adult offspring.^28–30^ Animal studies indicate that maternal high-fat or sugar diets during pregnancy reduce placental fatty acid transporter expression, impairing nutrient delivery and limiting fetal growth, while also disrupting fetal fatty acid synthesis, heightening offspring susceptibility to diet-induced non-alcoholic fatty liver disease (NAFLD) and obesity across developmental stages.^31–33^ Further research suggested that a high salt diet during pregnancy impairs mitochondrial function in the brains of offspring, thereby compromising offspring neurometabolic programming.^34^ More alarmingly, these adverse effects in mitochondrial function may persist across three generations,^35^ highlighting the detrimental impacts resulting from urbanized diet may be indelible. Similarly, these dietary patterns during pregnancy elevate the expression of pro-inflammatory cytokines such as interleukin (IL)-6, IL-1β, and tumor necrosis factor-alpha (TNF-α), increasing neonatal susceptibility to intestinal inflammation after birth.^3^ Moreover, consumption of high-fat diet (HFD) during pregnancy and lactation period has also been shown to disrupt the N-methyl-D-aspartate (NMDA) receptor expression in the brain of offspring after birth, leading to memory and behavioral disorders such as autism in rodent model,^36^ which can also persist into adulthood even after offspring dietary modifications.^37^ These indicate that maternal diets high in sugar and fat during pregnancy can have multifaceted effects on the health of the offspring. Furthermore, both developed and developing countries are experiencing hidden hunger, characterized by multiple micronutrient deficiencies, particularly iron, selenium, vitamin E.^38,39^ Deficiencies in folic acid, vitamin E, vitamin D, iron, and selenium have long been linked to placental aging, placental abruption, premature birth and even abortion,^40–43^ and some were found to be positively associated with offspring asthma, infantile eczema and increased the risk of autism spectrum disorder.^44–47^ The underlying mechanism may be associated with impaired mitochondrial function in the placenta.^48^ For instance, Phillips et al. demonstrated that vitamin D intervention mitigates mitochondrial dysregulation in placentas of obese women, thus reducing inflammation and suppressing adverse pregnancy outcomes.^49^ Furthermore, an alternative molecular mechanism proposes that micronutrient deficiencies may epigenetically alter fetal programming, ultimately influencing long-term health outcomes.^50^ A large-scale Finnish cohort study demonstrated that maternal vitamin D deficiency disrupts DNA methylation patterns in imprinted regions of somatic and germline cells, with intergenerational epigenetic implications that may influence brain, skeletal, cardiovascular, immune, and reproductive health of the offspring.^50^ Similarly, vitamin B_12_ has been linked to DNA methylation at cytosine-phosphate-guanine sites in both pregnant women and neonates, correlating with birth weight and cognitive outcomes in childhood.^51^ This evidence conveys the message that micronutrient deficiencies can influence maternal mitochondrial function and epigenetic modifications, thereby affecting offspring health.

Additionally, research is increasingly highlighting the potential health risks related to maternal exposure to ultra-processed foods. Several meta-analyses of cohort studies indicate that ultra-processed food intake during pregnancy is associated with higher rates of pregnancy preeclampsia, gestational diabetes and obesity,^52,53^ lower skeletal components of fetal growth and low birth weight,^54^ and increased risk of later-onset obesity and metabolic disorders, even NAFLD in offspring.^55–57^ The consumption of ultra-processed foods not only increases the intake of calories, salt, and sugar but may also exacerbate the consumption of food additives, resulting in various unfavorable effects.^58^ For example, in the mouse model, maternal intake of dietary emulsifiers during pregnancy and lactation can cause metabolic and cognitive deficits in offspring after birth,^59^ while consuming fried foods in pregnancy may expose them to neurodevelopmental impairments due to the production of neurotoxic byproduct acrylamide.^60^ Moreover, in observational studies of pregnant women-children pairs, high intake of artificial sweeteners such as aspartame and sucralose during pregnancy was associated with low-grade systemic inflammation^61^ and metabolic disorders in newborns,^62^ while animal models demonstrate that such exposure increases the risk of obesity later in life.^63^ Furthermore, exposure to phthalates and perfluoroalkyl compounds in food packaging materials during pregnancy is also associated with shorter anogenital distance in baby boys.^64,65^ The above evidence suggests that maternal consumption of ultra-processed foods can also affect the offspring health. However, current research primarily relies on animal models to investigate the specific effects of food additives and other food ingredients within contemporary urban diets during pregnancy and lactation. The limited human studies conducted to date are primarily observational and do not comprehensively explore the underlying mechanisms.

Moreover, environmental pollution associated with urbanization also emerges as a critical determinant of early developmental trajectories. A cohort study found that exposure to environmental particles (PM_2.5_ and ozone) during the first trimester increases the risk of preeclampsia, gestational hypertension, preterm birth,^66^ and even spontaneous abortion and stillbirth^67^; while exposure during late pregnancy significantly elevates the risk of attention-deficit/hyperactivity disorder (ADHD) in early childhood.^68^ Similarly, exposure to microplastics during pregnancy in humans has been shown to affect pregnancy outcomes, including fetal growth restriction.^69^ In addition, increased children’s exposures to urban-associated environments, characterized by particulate matter, indoor pest allergens, and mold, trigger systemic inflammatory responses and childhood asthma development.^70,71^ These highlight the critical role of urban environmental quality, both during the prenatal and postnatal periods, in early-life programming and its potential long-term impact on health outcomes.

Alongside maternal diet and environmental exposures during pregnancy, paternal factors also exert a substantial influence on offspring health. Studies have shown that a short-term high-fat diet in male mice leads to mitochondrial dysfunction in sperms, with sperm mitochondrial RNA being transmitted to the offspring, influencing their metabolic pathways and increasing the risk of glucose intolerance and insulin resistance.^72^ These findings suggest that paternal diet can mediate epigenetic alterations that impact offspring health. Moreover, paternal malnutrition-induced dysbiosis of the gut microbiome has been shown to affect maternal placental function, thereby influencing fetal development and leading to abnormal birth weight and reduced offspring survival.^73^ Notably, these effects are reversible by restoring the paternal gut microbiome before conception, further confirming the epigenetic influence of paternal factors on offspring health.

### Urban maternal nutrition behaviors contribute to gut microbiota assembly in early life

Urbanization not only affects the health outcomes of offspring but also profoundly influences the establishment of the infant gut microbiome during early life. Research has highlighted significant disparities in gut microbiota composition between infants born in urban and rural environments.^74–76^ For example, observational studies have identified urban infants at 1 year age to 6, exhibit a unique enterotype with a higher ratio of *Bacteroides* to *Prevotella* genera and a reduced microbial diversity compared to their rural counterparts, which may be associated with increased prevalence of atopic diseases in infants including asthma, autism, allergic rhinitis, and food sensitization.^16,75,77^ Furthermore, Dhakal et al. revealed that transplantation of rural infant microbiota into germ-free piglets enhances conventional dendritic cell induction in the small intestine compared to urban microbiota, suggesting rural microbiota mediated mucosal immune development toward enhanced tolerance against allergic conditions and pathogens.^77^ These studies highlight that urbanization-induced modifications profoundly alter gut microbiota composition and may be linked to the pathogenesis of immune-related disorders in offspring.

Although various factors may underlie the rural-urban differences in infant gut microbiota, maternal diet is one of major determinants. Maternal consumption of high-fat, high-sugar, high-salt and low-fiber diets in primate and rodent models has been shown to reduce the abundance of *Bifidobacterium* and *Lactobacillus* genera in offspring, linked to an increased risk of autism and obesity in offspring.^78,79^ These alterations are consistent with that observed in the above urban infants, indicating the crucial role of urbanized diet practice in mothers in shaping a distinct gut enterotype in urban infants. Additionally, food-grade titanium dioxide (TiO_2_) particles can be translocated into the placenta and meconium through transplacental passage to the fetus, resulting in a disturbance in glucose metabolism and gut microbiota in male offspring, such as an increase in β-diversity of gut microbiota and *Firmicutes*/*Bacteroidetes* ratio.^80^ However, beyond animal studies and observational human studies, direct evidence to establish a causal relationship between maternal diet, microbiome change, and early-life disease is limited due to the challenge of conducting clinical intervention studies in pregnant women.

## Mechanisms of maternal consumption of urbanized diet influence infant’s health in association with gut microbiota

Although the detrimental effects of maternal urbanized dietary practices on the offspring health and gut microbiota assembly are increasingly recognized, the underlying mechanisms remain far from clear. Emerging evidence shows that urban dietary patterns significantly alter gut microbiota composition and metabolic profiles in mother, potentially disrupting the host’s immune and metabolic systems.^6,81^ These microbiota dysbiosis, metabolic abnormalities, and immune dysfunctions effects resulting from urbanized diet may transmit from the mother to offspring via placental transfer or breast milk, further adversely affecting offspring health.^82,83^ Therefore, we synthesize current research to clarify how specific gut microbiota, key metabolites, and immune-related molecules associated with urbanized diets are transferred from the mother to offspring and how these factors further affect offspring health.

## Modulation of prenatal urbanized diet on the maternal-fetal crosstalk

### Maternal-fetal transmission of microbial metabolites

**Figure 2.**
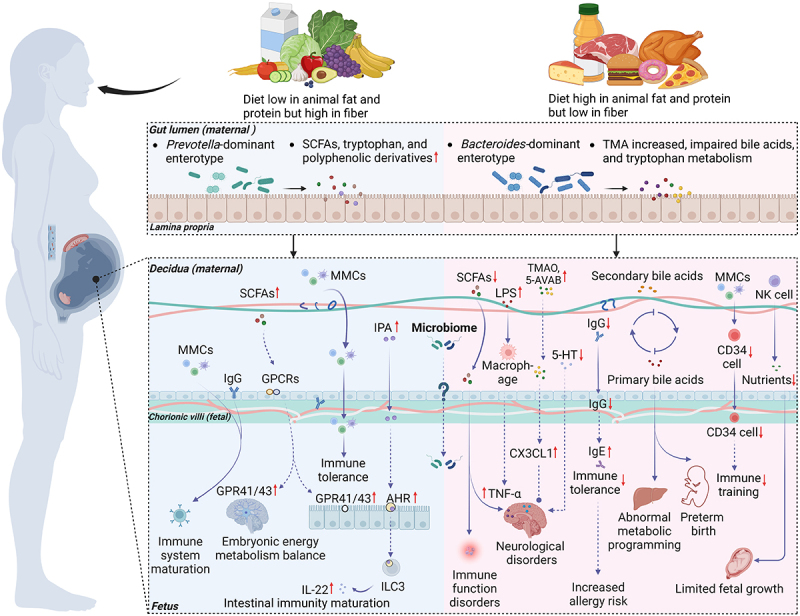
Mechanisms by which prenatal urbanized diets affect maternal-fetal crosstalk. Effects of healthy (left) and urbanized (right) prenatal diets on fetal development are illustrated. Urbanized dietary patterns significantly alter gut microbiota composition and metabolic profiles, potentially disrupting the host’s immune and metabolic systems. These microbiota dysbiosis, metabolic abnormalities, and immune dysfunctions resulting from urbanized diets may transmit from the mother to offspring via placental transfer, further adversely affecting offspring health. Human and animal studies were distinguished by using solid and dashed lines, respectively. SCFAs, short-chain fatty acids; TMA, trimethylamine; TMAO, trimethylamine N-oxide; 5-AVAB, 5-aminovaleric acid betaine; IPA, 3-indolepropionic acid; 5-HT, serotonin; LPS, lipopolysaccharides; MMCs, maternal microchimeric cells; GPCRs, G protein-coupled receptors; IgG, immunoglobulin G; AHR, aryl hydrocarbon receptor; IL-22, interleukin-22; ILC3, intestinal group 3 innate lymphoid cells; tnf-a, tumor necrosis factor-alpha; CX3CL1, chemokine C-X3-C motif ligand 1; NK cell, natural killer cell.

Numerous pieces of evidence demonstrate that the transition in diet patterns from agricultural to industrial or post-industrial societies leads to profound alterations in gut microbiota, including a shift from a *Prevotella*-dominated enterotype to a *Bacteroides*-dominated enterotype.^84,85^ These changes are particularly crucial during pregnancy, as they not only affect maternal health but also have long-term implications for offspring development. In light of these implications, emerging research has begun to investigate the impact of maternal diet on gut microbiota composition during this critical gestational period. For example, similar shifts in microbiota composition have been observed in pregnant women, with adherence to a Mediterranean diet maintaining a *Prevotella*-dominant enterotype,^86^ while an increased abundance of *Collinsella* in the maternal gut microbiota under Western diet.^87^ Though these findings are derived from correlation analyses in cohort studies, they offer compelling evidence suggesting that urbanized dietary patterns may exert a significant influence on maternal gut microbiota. More importantly, these shifts in microbiome composition coincide with impaired capacities for metabolite production, such as SCFAs, polyphenol metabolites and tryptophan catabolites, and bile acid derivatives.^6,88,89^ Evidence indicates these substances may traverse the placenta to the fetus, playing a crucial role in fetal growth and development (Figure 2).^90,91^ For example, some tryptophan metabolites derived from maternal microbiota such as 3-indolepropionic acid (IPA) have been detected in umbilical cord blood.^92^ These tryptophan metabolites can function as typical aryl hydrocarbon receptor (AHR) ligands, which are essential for the differentiation of intestinal group 3 innate lymphoid cells (ILC3s) to produce IL-22. This process regulates immune response and the intestinal immunity maturation in fetus.^93–95^ Other tryptophan metabolites, like serotonin (5-HT), are present in the fetal brain to support its development and reduce the risk of neuropsychiatric disorders later in life.^96^ Experiments with germ-free mice confirmed that the presence of gut microbiota in dams can increase tryptophan metabolite levels in the fetal gut-brain axis,^95^ highlighting the importance of maternal microbiota in fetal neurodevelopment. However, a maternal HFD during pregnancy can lower 5-HT levels in the fetal brain.^96^ These findings suggest that maternal Western dietary patterns may reduce the production of 5-HT and other tryptophan metabolites in the gut, limiting their transfer across the maternal-fetal interface, ultimately leading to negative impacts on fetal development.

Besides, trimethylamine N-oxide (TMAO), another important microbial metabolite that may transfer from mother to infant, modulates immune responses by inhibiting neutrophil extracellular trap formation, potentially protecting the placenta in cases of gestational diabetes mellitus and supporting fetal development.^97^ However, it also showed that TMAO may upregulate chemokines chemokine C-X3-C motif ligand 1 (CX3CL1) in fetal brain, potentially disrupting various neuropathological processes.^98^ Other trimethylated compounds, such as 5-aminovaleric acid betaine (5-AVAB), may exert similar effects.^91,99^ However, studies have shown that urbanized dietary patterns rich in animal products lead to elevated TMAO levels in adults, contributing to multiple diseases, such as cardiovascular disorders and diabetes.^100–102^ Whether such dietary patterns during pregnancy will lead to excessive TMAO accumulation in fetal and adversely affect fetal development warrants further exploration.

Another significant characteristic of urban dietary patterns is the reduced intake of plant-based foods, which directly leads to a notable decrease in the levels of polyphenolic derivatives and SCFAs, both of which are crucial for promoting fetal brain development. Specifically, polyphenolic derivatives, such as hydroxyphenyl acetic and hydroxyphenyl, have been shown to interfere with the assembly of beta-amyloid peptides and thus improve brain health.^103^ Similarly, SCFAs are involved in brain development during the embryonic stage via the G protein-coupled receptor (GPCR) signaling pathways, including GPR41/43 receptors, which are also expressed in intestinal epithelium to promote gut health.^104^ Therefore, the decreased consumption of plant-based food, which leads to reduced production of beneficial metabolites, may partially contribute to the adverse impact of urbanized maternal dietary patterns on fetal brain development. Besides, the decline in SCFAs affects the activation of GPCRs, which may also potentially influence fetal immune function and energy metabolism, thereby increasing their risk of food allergies and obesity in childhood.^105,106^ Recent animal studies indicate that supplementation with fiber such as inulin that promotes SCFAs production can reduce the risk of obesity in adult offspring born to mothers who consumed a Western diet during pregnancy.^104^ This further provides evidence that SCFAs serve as key mediators of the impact of maternal diets on the health of offspring. Furthermore, maternal diets during pregnancy can impact fetal growth by mediating bile acid metabolism. The fetus lacks an intrinsic bile acid circulation system and therefore relies on the mother for this process. This involves the transfer of primary bile acids from the fetus to the mother, where they are converted into secondary bile acids by the maternal gut microbiota, and then transported back to the fetus via the placenta, forming a distinct fetal-placental-maternal bile acid cycle.^107,108^ Excessive dietary fat (>35% of total daily caloric intake from fat)^109^ intake during pregnancy elevates total bile acid levels in maternal serum, disrupting the balance of primary and secondary bile acids, altering the gut microbiota, and consequently impacting fetal-placental-maternal bile acid circulation. Disruption of this circulation balance leads to abnormal fetal cardiac and hepatic metabolic programming,^110,111^ even accompanied by adverse pregnant outcomes, such as preterm birth.^108^ These pieces of evidence suggest that an urbanized diet may disrupt the maternal production of some key microbial metabolites, which could be one of the underlying mechanisms for adverse offspring outcomes. Notably, the hypothesis that these metabolites can traverse the placenta is based on the fact that they are derived from gut microbiota while the fetus is relatively sterile. However, there is a lack of robust evidence demonstrating that these metabolites can be transmitted from the mother to infant through isotope-labeling technology. Furthermore, aside from SCFAs, there is limited evidence confirming the causal relationship or mechanism of action between other metabolites and fetal development through maternal supplementation.

### Maternal-fetal transmission of microbial macromolecules

In addition to small molecule metabolites, macromolecular components of maternal gut microbiota, such as bacterial lipopolysaccharides (LPS), cell wall peptidoglycan, and extracellular vesicles, have also been detected in utero.^112–114^ Research has revealed that maternal HFD during pregnancy can lead to impaired intestinal barrier function in mothers, subsequently elevating circulating levels of LPS in the maternal bloodstream.^115^ Further studies demonstrated that fluorescently labeled LPS infused to dams can be detected in the uterus, fetal membranes, and placenta, suggesting potential transmission of such molecule to fetus,^116^ where it triggers a pro-inflammatory response characterized by elevated TNF-α, IL-1β, and IL-6 levels in the developing brain, leading to brain inflammation and ultimately contributing to adult neurological disorders.^117^ However, as these findings primarily stem from LPS infusion models, they may not fully recapitulate the chronic and dynamic nature of in-utero exposure during human pregnancy.

While both gut microbial metabolites and macromolecules can traverse the placental barrier to reach the fetus, it remains controversial whether a whole microbial cell from the mother can cross the placental barrier and subsequently colonize and survive in the intrauterine environment. Recent sequencing-based studies have detected bacterial genera such as *Lactobacillus*, *Staphylococcus*, *Streptococcus*, *Enterococcus*, *Bifidobacterium*, *Prevotella*, *Micrococcus*, and *Finegoldia* genera in fetal intestinal contents.^118–120^ The presence of *Micrococcus luteus* in the fetal microbiome is associated with distinct epithelial transcriptomic patterns and T-cell composition, implicating its involvement in immune imprinting.^118^ Moreover, Mishra et al. demonstrated that intrauterine exposure to viable bacteria shapes fetal immune cell development in a cell-microbiota co-culturing model, further supporting the notion that microbial signals contribute to immune programming in early life.^119^ This evidence supports the notion of viable bacterial colonization within the uterine environment, indicating that intrauterine microbes may exist and influence early fetal growth and development. However, a contentious debate within the academic community asserts that the microbiological signals detected in the intrauterine environment likely result from contamination during sample collection, culture processes, DNA extraction, and sequencing.^121^ Therefore, rigorous contamination-removal strategies are essential for accurately characterizing microbial presence. However, the lack of standardized protocols for sampling and analysis introduces biases that can lead to false findings. Addressing these technical challenges is crucial for determining whether and how maternal urbanized dietary patterns influence microbial transmission to the fetus and shape early-life microbial priming, ultimately informing strategies to promote fetal health and development.

### Maternal-fetal transmission of immune elements

The transfer of maternal-derived immune factors via the placenta, including immune cells, antibodies, cytokines, and chemokines, is crucial for modulating fetal immune development, promoting its maturation, and preparing it to respond to postnatal pathogens and other immune challenges.^122,123^ An excessive response to these non-self elements may compromise immune tolerance mechanisms, leading to a breakdown of immune homeostasis and an increased susceptibility to autoimmune pathologies such as multiple sclerosis, systemic lupus erythematosus, and type 1 diabetes.^123^ However, insufficient or disrupted transfer of maternal immune factors can compromise fetal immune training, leading to delayed or incomplete immune system maturation and reduced capacity to combat postnatal pathogens.^124^ Studies have shown that maternal obesity induced by a high fat/sugar diet during gestation reduces the quantity of CD34^+^ progenitor cells and natural killer (NK) cells, as well as elevated CD4^+^ T cells and CD8^+^ T_reg_ cells in cord blood, subsequently diminishing the number of these immune cells transmitted to the fetus and impairing early-life immune training.^125,126^ Consequently, this may alter fetal development and their capacity to resist various forms of immune system damage.^127^ Moreover, HFD may lead to maternal chronic low-grade inflammation, characterized by elevated serum levels of inflammatory cytokines such as TNF-α, interferon-gamma (IFN-γ), and IL-6.^128^ These proinflammatory cytokines, particularly T helper type 1 (T_H_1) cytokines such as TNF-α, which can pass through the placental – fetal barrier and reach the fetal brain, may induce excessive immune tolerance and impact neurodevelopment and cognitive performance in offspring.^129,130^ Besides, non-human primate model demonstrated that maternal Western-style diet exposure during pregnancy alters long-term developmental programming in hematopoietic stem and progenitor cells, leading to persistent pro-inflammatory phenotypes in both fetal and juvenile offspring.^28^ The above evidence illustrates that urbanized dietary patterns during pregnancy exert detrimental effects on the programming of the fetal immune system in utero.

Furthermore, diet-induced alterations in gut microbiota can shift maternal immunoglobulin G (IgG) responses, which can cross the placenta by attaching to the Fc receptor (FcRn) expressed in syncytiotrophoblast, thereby protecting neonate from infection in the first weeks of life.^131^ Allergen-specific IgG in maternal cord blood may also be transferred to the next generation, preventing immune tolerance and protecting the children from being sensitized to the allergen.^132^ Research shows maternal consumption of HFD during pregnancy is associated with lower IgG levels in offspring, further compromising their resistance to allergies.^133^ In contrast, phytosterols and soluble dietary fibers are positively associated with IgG3 and IgG2 levels in cord plasma respectively,^134^ which can cross the placenta and potentially reduce fetal food-specific IgE levels, thereby decreasing the risk of allergies in offspring.^135^ Therefore, elucidating the production of different antibodies by various food ingredients in maternal diets and its impact on early-life immune system development will be crucial in preventing the rising incidence of autoimmune diseases, including allergies, asthma, and other related conditions.

## Modulation of postnatal urbanized diets on the maternal-infant crosstalk via breast milk

**Figure 3.**
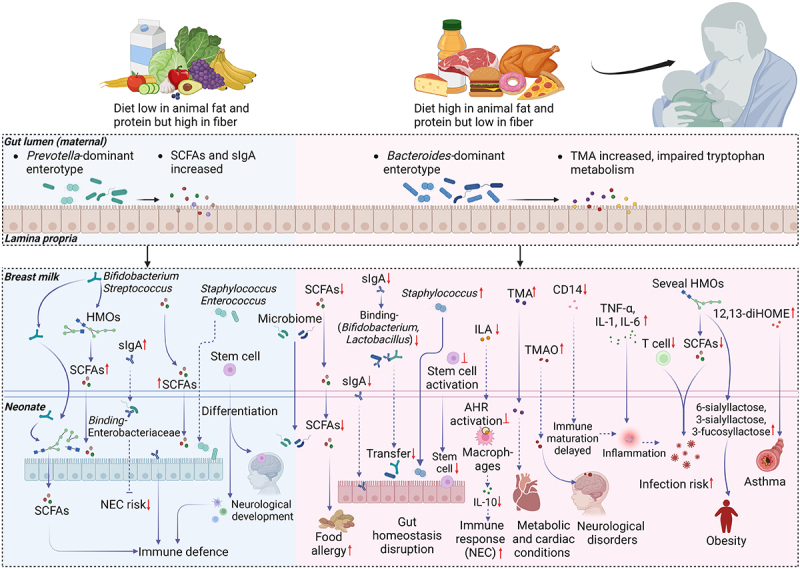
Mechanisms by which urbanized postnatal diets affect the maternal-infant crosstalk. Effects of healthy (left) and urbanized (right) postnatal diets on neonatal development are illustrated. Urbanized diets alter the composition of HMOs, immune cells, microbiome (bacteria, fungi, and viruses), and metabolites in breast milk, which can be transferred to offspring, thus influencing the assembly of the early-life gut microbiome and the development of the immune system. Human and animal studies were distinguished by using solid and dashed lines, respectively. SCFAs, short-chain fatty acids; TMA, trimethylamine; TMAO, trimethylamine N-oxide; HMOs, human milk oligosaccharides; 5-AVAB, 5-aminovaleric acid betaine; ILA, indole-3-lactic acid; 12,13-diHOME, 12,13-dihydroxy-9Z-octadecenoic acid; sIgA, secretory immunoglobulin A; AHR, aryl hydrocarbon receptor; IL-6, interleukin-6; TNF-α, tumor necrosis factor-alpha; NEC, necrotizing enterocolitis.

During the postpartum lactation period, breast milk plays a crucial role in nurturing the connection between mother and infant, serving as the primary source of nutrition for newborns in their early stages of development. Beyond providing essential macronutrients and micronutrients, breast milk contains a variety of bioactive macromolecules, oligosaccharides, immune cells, and microbiota.^136^ These components can be transferred to the infants, significantly influencing the neonatal immune system, metabolic processes, and neurological development during early life.^136,137^ Current data predominantly suggests that maternal diets significantly affect the composition of these nutritional and biological elements, as well as the microbiota present in breast milk (Figure 3). Therefore, we summarize current findings to assess how variations in maternal urbanized diet influence breast milk composition and consequently infant development.

### Maternal-infant transmission of human milk oligosaccharides

Human milk oligosaccharides (HMOs), unconjugated complex glycans abundantly present in human milk, serve as a primary nutritional source for newborns and exert profound effects on offspring health by fostering a balanced gut microbiota, enhancing immune responses, and reducing the risk of infections and allergic conditions.^138^ The mechanism underlying these effects lies in the ability of HMOs to support the growth of beneficial bacteria in infant such as *Bifidobacterium* spp., thereby facilitating the establishment of a gut niche during early life.^139^ Moreover, the breakdown of HMOs by microbe produces secondary metabolites such as SCFAs, which are essential for the development and modulation of immune and intestinal systems in infants.^140^ HMOs are composed of three primary types: fucosylated HMOs, sialylated HMOs. and neutral core HMOs, and different types can be degraded by various gut bacteria to exert diverse physiological functions.^141,142^ Although the basic structure of HMOs is primarily determined by maternal genetic factors, research suggests that maternal dietary habits can also influence the composition and diversity of HMOs.^143^ A previous study has revealed that maternal obesity is associated with several higher concentrations of fucosylated and sialylated HMOs, such as 6-sialyllactose, 3-sialyllactose, and 3-fucosyllactose, leading to increased risk of infant adiposity.^144^ A galactose-rich diet increases the concentration of fucosylated HMOs, which drives the expansion of a prevalent *Streptococcus* genus that possesses annotated fucosidase genes in human milk, thereby facilitating their colonization in infants.^145^ In contrast, a diet high in fats and carbohydrates leads to an increase in *Staphylococcus* genus in breast milk and a reduction in the concentration of sialylated HMOs.^145^ Moreover, intervention with a Mediterranean dietary pattern in obese mothers results in a reduction of total HMOs concentrations, particularly fucosylated HMOs (lacto-N-fucopentaose-III and difucosyllacto-N-tetrose), demonstrating positive effects in preventing infant obesity.^146^ These observed modifications indicate that urbanized dietary pattern during lactation may alter HMOs composition, thereby impacting early-life nutritional programming and developmental trajectories. However, current studies have only established correlations between some dietary patterns/specific dietary components and HMOs types; the health consequences and the mechanism behind such changes in HMOs composition warrant further exploration.

### Maternal-infant transmission of immunological components

Breast milk also functions as a natural immunological reservoir, providing immune components such as cytokine, antibodies, and immune cells that facilitate the education and maturation of the neonatal immune system, thereby enhancing the capacity of neonatal to resist infections caused by pathogens.^136^ For example, secretory IgA (sIgA) in breast milk can also localize in the infant gut, binding to pathogens such as some species from Enterobacteriaceae family, effectively preventing their adhesion to and infiltration of mucosal epithelial cells, and thereby reducing the risk of preterm necrotizing enterocolitis (NEC).^147,148^ sIgA also binds to beneficial bacteria found in breast milk such as members from *Bifidobacterium*, *Streptococcus,* and *Lactobacillus* genera, facilitating their transfer from breast milk to the infant, and subsequently assisting in the establishment of the early-life intestinal microbiota.^149,150^ This process is crucial for enhancing the local innate immune response.^151^ However, maternal HFD, malnutrition, and vitamin A deficiency have been demonstrated to reduce sIgA in the small intestine, adversely affecting the transport efficiency of sIgA to the mammary glands, consequently decreasing the amount available to neonates through breast milk.^152,153^ These suggest that influences of urbanized dietary practice on maternal sIgA levels may impact the infant’s susceptibility to infections and overall immune function. Furthermore, some immune cells such as stem cells present in breast milk can reach the intestines of newborns and migrate to various organs, such as the brain, where they differentiate into different cells, promoting the development of the newborn’s immune and neurological systems.^154,155^ Likewise, pre-conception infection of the mother will facilitate the transfer of T_H_2-competent CD4 T cells to offspring via breast milk to localize in the digestive tract and spleen, thereby conferring long-lasting protective immunity against infection.^156,157^ Compared with Mediterranean and high-fiber diets, the Western diet significantly elevates the risk of mastitis and obesity in mothers, and induces a high level of TNF-α, IL-1β, IL-6, and B lymphocytes in breast milk, concomitant with a reduction in both the quantity and proliferative capacity of T lymphocytes and T CD4^+^ cells as well as secretory CD14, and even extends to the suppression of stem cell functionality in breast milk.^158–161^ However, the extent to which urbanized diets modify these immune components and the resulting health consequences for the offspring are still not fully understood.

### Maternal-infant transmission microbes and microbial metabolites

In addition to immune components, such as antibodies or cells, and essential nutrients, breast milk also harbors a diverse microbiota, including genera such as *Staphylococcus*, *Streptococcus*, *Enterobacter*, and *Bifidobacterium*,^162,163^ which serves as primary sources for infant microbiota assembly. Emerging evidence suggests that dietary patterns significantly influence the composition of microbiota in breast milk.^164^ For example, human studies have revealed that the intake of polyunsaturated fatty acid, and plant protein and fiber is positively correlative with the level of *Lactobacillus* and *Bifidobacterium* genera in breast milk.^165,166^ Conversely, a diet high in animal protein and lipids is associated with an increased abundance of *Ruminococcus*, *Lachnospiraceae* and *Staphylococcus* genera, and decreased *Lactobacillus* spp. in breast milk.^164,167^ Moreover, prior to the introduction of complementary foods in infants, breast milk serves as the exclusive source for the acquisition of crucial microbial metabolites, including SCFAs and tryptophan derivatives. These metabolites play a pivotal role in early infant development.^168^

Animal studies further demonstrated that maternal consumption of a Western diet leads to a reduction in tryptophan metabolites such as indole-3-lactic acid (ILA), which can impede AHR signaling and reduce IL-10 expression, potentially preventing NEC.^169,170^ While in another mother-infant dyad, a significant negative association was observed between breast milk SCFAs and vegetable consumption, related to the later development of atopic eczema or food allergy.^166^ In addition to beneficial metabolites such as tryptophan derivatives, harmful metabolites resulting from diet-microbiota interactions, including TMAO in mice,^171^ and TMA^172^ and 12,13-dihydroxy-9Z-octadecenoic acid (12,13-diHOME) in human milk,^173^ have also been identified in breast milk. A cohort study found that TMAO levels are positively correlated with children’s vascular damage,^174^ while 12,13-diHOME, an oxidized linoleic acid metabolite produced by gut bacteria like *Lactobacillus* and *Streptococcus* genera,^175^ is strongly associated with disruptions in the gut microbiome during early life and with the development of atopy and asthma in childhood.^175^ The consumption of Westernized and ultra-processed diets may elevate the levels of these harmful metabolites in the gut or bloodstream. Therefore, elucidating how diet modulates the levels of these key metabolites in human milk can help us develop dietary recommendations for mothers to optimize essential nutritional support during early infant development.

Interestingly, diets not only alter the composition of the microbiota in breast milk but also influence the vaginal microbiota, which serves as another source for establishing the initial gut microbial community in newborns delivered vaginally. *Lactobacilli* genus are dominant in the maternal vaginal microbiota, and their transmission to infants is essential for establishing the initial gut microbial community of newborns.^176,177^ Maternal obesity induced by HFD leads to reduced microbial diversity^178^ and decreased *Lactobacillus* abundance, which is linked to excessive inflammation and preterm birth.^179^ By contrast, a high-starch diet (total carbohydrate and glycogen intake) may increase the glycogen content in the vagina. The additional glycogen can be utilized by *Lactobacilli* genus to produce lactic acid and other by-products, effectively lowering the vaginal pH. This, in turn, fosters an environment conducive to *Lactobacilli* genus proliferation, while simultaneously inhibiting the colonization of pathogens, thereby maintaining vaginal health.^180^ Besides, increased consumption of low-fat dairy products also enhances the relative abundance of these beneficial species,^181^ while the mechanism remains clear. These observations suggest that an urbanized diet rich in fiber and low in carbohydrates may curtail the growth of beneficial microbes, such as *Lactobacilli*, by creating an unfavorable vaginal environment, thereby hindering the colonization of these microbes in the infant gut.

### Maternal-infant crosstalk via fungi and viruses transmission

In addition to bacteria, the establishment of intestinal viruses and fungi is essential for developing a stable and healthy infant gut microbiome.^182,183^ Observational studies in humans have indicated a correlation between disturbances in the gut virome, characterized by decreased diversity and specific viral signatures, such as *Circoviridae*, and various early-life diseases, including NEC, malnutrition, and type 1 diabetes.^184–186^ For example, a longitudinal birth cohort showed the deficiency of certain temperate phages such as *Clostridiales*-infecting *Maisaviridae* and *Faecalibacterium*-infecting *Sofieviridae* at 1 year of age is linked to asthma. Such association is independent of their bacterial hosts.^187^ However, these findings remain hypothesis-generating and observational, requiring further confirmation. One postulated mechanism is that the gut virome is involved in the development of the gut mucosal immune system either indirectly (for example, through selective pressure on bacteria to modulate the sequence of bacterial colonization in early life)^188^ or directly (for example, through the recognition of viral pathogen-associated molecular patterns).^189^ Moreover, viruses occur transmission from the mother to infant, with approximately 15%-30% of the viral contigs in infants derived from the mothers.^190,191^ In this transmission process, maternal bacteriophages may shape the infant’s virome composition, thereby influencing the microbiota composition and immune system development.^192^ Moreover, although some maternal bacteria fail to colonize the infant, phages can transfer the genes from these bacteria to the infant, thereby further influence the assembly and metabolic potential of infant gut microbiota.^137,193^ This evidence indicates the crucial role of virome assembly in regulating bacterial colonization and health in early life and emphasizes the importance of maternal virome in such process. Diet is essential in shaping maternal gut virome, contributing to approximately 8% variation of gut virome.^194^ Notably, the urbanized dietary habit greatly affected the composition of gut virome, leading to a decrease in relative abundance of *Siphoviridae* and a notable rise in abundance of *Caudovirales*.^195,196^ Though limited studies suggested that milk lipids may protect phages during transmission from the mother to infant,^197^ it remains insufficient to clarify the impact of maternal diet on viral transmission. Therefore, whether this shift in viral community induced by urbanized diet can alter transmission patterns of viruses from the mother to offspring and thus lead to various diseases early in later life is warranted for further research.

Similarly, the significance of gut fungi in early-life health is also increasingly highlighted. Investigations in animal models have shown that fungi can produce synergistic interactions with bacteria, skew the immature immune system, and affect susceptibility to immune-mediated disorders in adulthood.^198^ A longitudinal study tracking the evolution of gut fungal community from 3 to 48 months in 888 children found the community was initially occupied by *Penicillium paneum* and a set of *Candida* species including *Candida albicans*, *Candida parapsilosis*, and *Candida zeylanoides*, and were then gradually predominated by *Saccharomyces cerevisiae*.^199^ The disruption in such evolution pattern is associated with various detrimental effects on infants. For instance, a human study showed that the abundance of *Saccharomyces* at three months and *Candida* at one year positively correlated with body mass index of infants after one year of age,^200^ indicating the possible link between fungal community maturation and growth trajectories in infants. Moreover, a reduction in α-diversity and the reduction of the *Saccharomyces cerevisiae* at one year old were positively associated with the occurrence of allergy in children at five years,^201^ indicating the colonization of *Saccharomyces cerevisiae* in early life may be a key contributor to later life health. These fungi are primarily derived from mothers before the introduction of solid food to infants. However, animal studies have found that urbanized dietary patterns lead to significant changes in the abundance of these fungi, such as a decrease in *Saccharomyces* ^202^ and an increase in *Candida*.^203^ Nevertheless, whether urbanized dietary patterns therefore affect the evolutionary pattern of gut fungal community and eventually impact the infant health is worth further exploration. Despite limited current evidence, it emphasizes the need for a better understanding of the underlying effects of urbanized diets in these underestimated components in maternal and infant guts. Addressing such questions will facilitate more rational designs of microbiome-directed dietary therapies specifically tailored for maternal and offspring health.

## Concluding remarks and future perspectives

The rise of NCDs over the past decades has spurred significant interest in the relationship between urbanized diets and gut microbiota, advancing microbiome-directed dietary therapies for disease management. Our review underscores that metabolic, immunological, and microbial changes induced by the interplay between urbanized diets and gut microbiota can be transmitted from the mother to offspring, compromising early-life and even later-life health. Despite our knowledge is far from complete, there is a compelling need to move beyond interventions upon disease onset in adulthood and into prevention at the very beginning of life through microbiome-directed dietary therapies for women during pregnancy and lactation.

To achieve this goal, identifying specific molecules such as SCFAs, TMAO, and so on, in the blood, or breast milk of mothers that determine the offspring health would be the first step. These molecules would serve as critical indicators of the metabolic and immunological alterations influenced by maternal diet and gut microbiota, thereby providing a scientific basis for proactive nutritional interventions. However, given the limited availability of well-defined longitudinal datasets for mother-infant dyads, the identification of biomarkers that significantly impact offspring health remains scarce. Furthermore, even though existing cohorts have identified several differential metabolites, their causal effects on long-term health trajectories and the underlying mechanisms are still far from being validated. Future research should prioritize the establishment of robust and diverse cohorts that follow mother-infant pairs over extended periods, capturing a comprehensive range of data, including dietary habits, microbial profiles, metabolomes, and various health metrics, to identify biomarkers in mothers indicating risks for offspring diseases and to validate the mechanisms underlying these associations.

**Figure 4.**
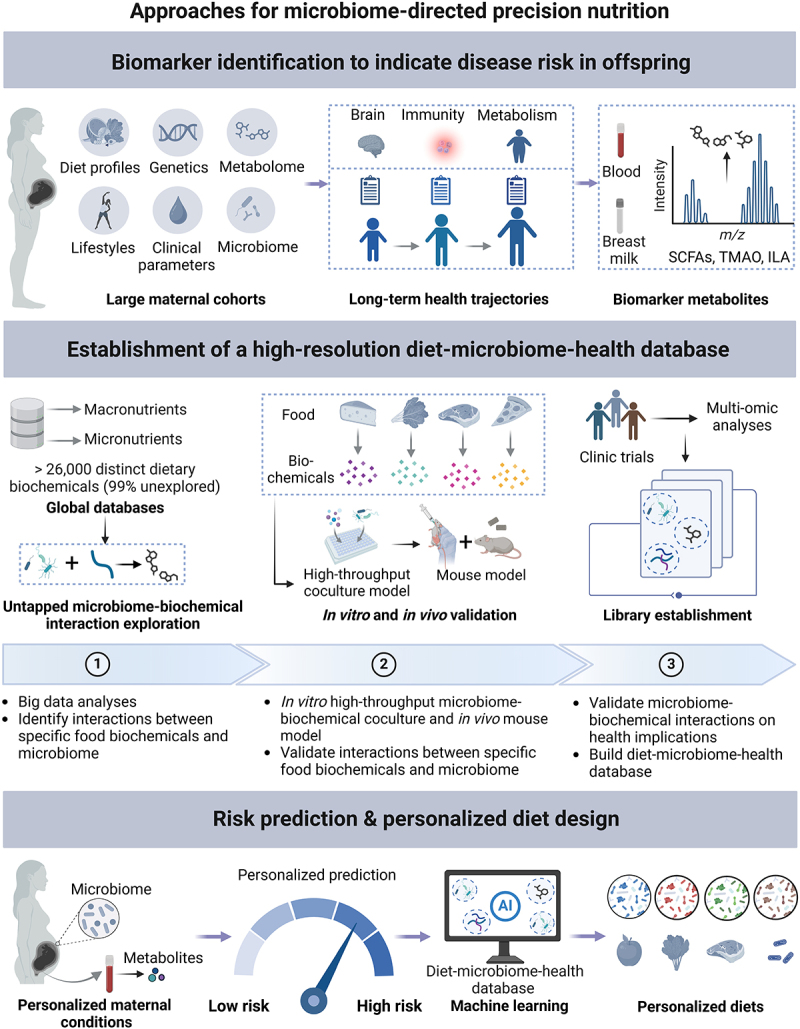
A proposed framework for the development of microbiome-directed precision diet. The framework consists of a three-step strategy: 1) identify specific molecules in the blood, or breast milk of mothers to predict the long-term health trajectories of offspring; 2) establish a comprehensive database that captures information on human and gut microbial metabolism and their links to hundreds of diseases and nutritional data, aiming to predict host-diet-microbiome interactions and their metabolic output; 3) predict disease risks in their offspring by measuring pregnant women’s metabolic profiles and customizing dietary and nutrition strategies to modulate key metabolite levels according to each pregnant woman ’s unique gut microbiome composition and function. Continuous optimization of algorithms and databases is essential to improve the ability to predict the host and microbial metabolic output of a defined diet, ultimately maximizing maternal nutritional benefits and enhancing offspring health.

Deciphering the mechanisms by which specific dietary components regulate the gut microbiota to produce these biomarker metabolites would be the next step. A comprehensive understanding of these processes will enable the development of targeted dietary interventions aimed at modulating biomarkers in maternal blood and breast milk, thereby potentially improving maternal and children’s health outcomes. Nonetheless, inter-individual human genetic and gut microbiota variability will lead to heterogeneous responses to a defined diet, complicating the ability to develop an effective population-based dietary intervention.^204,205^ Therefore, future research needs to move beyond a one-size-fits-all dietary guideline to effective and personalized health recommendations by predicting the metabolic outputs of various dietary components based on each individual’s genome, microbiome, and blood parameters. To advance this goal, studies have made significant efforts to unravel these intricate relationships between diet, gut microbiota, and host. Several databases have been established, such as FooDB,^206^ United States Department of Agriculture (USDA) FoodData,^207^ Virtual Metabolic Human,^208^ and AGORA2,^209^ which provide detailed information on food components, human, and gut microbial metabolic capacities. These databases, particularly when combined with genome-scale metabolic models, provide the possibility of predicting host responses to diets and gut microbial metabolic outputs based on microbiome-centric multi-omics data. This has been leveraged to design personalized dietary, prebiotic, and probiotic interventions, which have shown promising results in regulating glucose levels.^204,210^ However, current databases are far from complete, as previous research has primarily focused on macronutrients (fats, proteins, and carbohydrates, especially plant polysaccharides). Diets also contain a vast array of micronutrients (more than 26,000, e.g. polyphenols, lignans) and distinctive components of modern diets (such as food additives and Maillard reaction byproducts from excessive processing), whose interplay with gut microbiota remains largely unknown.^211^ Further *in vitro* (high-throughput microbiome-food coculture), and *in vivo* (defined microbiome colonization and food administration) experiments, and clinical trials (specific component) are demanded to determine and validate the responsiveness of different gut microbiota to these understudied food components and the health effects. Addressing these gaps will complete current database of diet-gut microbiota-host triads and enhance the predictive power of existing models and improve the efficacy of personalized dietary interventions (Figure 4).

Moreover, expanding the implementation of precision nutrition to improve maternal and child health poses even greater challenges, given the sensitivity of pregnant women and infants to new dietary supplements and microbial interventions. Furthermore, key questions for future research are summarized in Box 1. Continued efforts to refine personalized approaches and validate them through rigorous trials will be vital to ensure safety and efficacy, ultimately paving the way for more precise and beneficial health outcomes in these vulnerable populations.

**Table:** 

Box 1 Top Questions for Future Research
**Limited evidence from human studies** The long-term epigenetic effects and health consequences of maternal diet remain unclear due to the lack of longitudinal cohort studies across lifespan. Rigorous clinical controlled trials are lacking in delineating the specific effects of individual dietary components and in establishing causal relationships. Compared to high-fat diet, dietary fiber, and vitamins, there is a relative paucity of evidence from human and animal studies regarding the detrimental effects of food additives and other harmful components in ultra-processed foods consumed by mothers on their offspring. **Unclear mechanisms during the prenatal stage** Despite the identification of certain microbial metabolites that may be transmitted in utero in current literature, there is a lack of definitive techniques, such as fluorescent labeling, to verify their intrauterine transfer. The precise mechanisms by which these metabolites influence fetal development remain to be elucidated. The impact of urbanized dietary patterns on the intrauterine transfer of maternal immune cells and components remains unclear, and the underlying mechanisms as well as the corresponding consequences require further investigation. It remains to be elucidated whether urbanized diet-driven gut microbiota modifications can be vertically transmitted to offspring through viable bacteria from the mother. **Unclear mechanisms during the postnatal stage** Urbanized diet can modify the composition of maternal human milk oligosaccharides (HMOs), while the underlying mechanisms remain unclear. Additionally, the consequences of different HMO types for offspring are not well understood. Apart from a few metabolites (such as tryptophan), the roles of other microbial metabolites in human milk for infant growth and development remain unclear. The effects of urbanized dietary patterns on immune components in human milk, along with the underlying mechanisms and consequences, require further investigation. The origin of the human milk microbiome remains elusive and the effects of maternal diet on its composition are not yet well elucidated.

## Data Availability

The estimated prevalence data of NCDs are available for download from the Global Burden of Disease Study (GBD) 2021 (https://vizhub.healthdata.org/gbd-compare/).
